# Neighborhood disadvantage and biological aging biomarkers among breast cancer patients

**DOI:** 10.1038/s41598-022-15260-0

**Published:** 2022-06-30

**Authors:** Jie Shen, Bernard F. Fuemmeler, Vanessa B. Sheppard, Harry D. Bear, Renduo Song, Wong-Ho Chow, Hua Zhao

**Affiliations:** 1 Departments of Family Medicine and Population Health, School of Medicine, Virginia Commonwealth University, Richmond, VA 23284 USA; 2 Health Behavior and Policy, School of Medicine, Virginia Commonwealth University, Richmond, VA 23284 USA; 3 Department of Surgery, School of Medicine, Virginia Commonwealth University, Richmond, VA 23284 USA; 4 Departments of Epidemiology, The University of Texas MD Anderson Cancer Center, Houston, TX 77030 USA

**Keywords:** Cancer, Biomarkers, Risk factors

## Abstract

Living in a disadvantaged neighborhood is associated with adverse clinical outcomes among breast cancer patients, but the underlying pathway is still unclear. Limited evidence has suggested that accelerated biological aging may play an important role. In this study, using a sub-sample of 906 women with newly diagnosed breast cancer at M.D. Anderson, we examined whether levels of selected markers of biological aging (e.g., allostatic load, telomere length, and global DNA methylation) were affected by neighborhood disadvantage. The Area Deprivation Index was used to determine the neighborhood disadvantage. Based on the median ADI at the national level, the study population was divided into low and high ADI groups. Overall, breast cancer patients from the high ADI group were more likely to be younger and non-Hispanic Black than those from the low ADI group (*P* < 0.001, respectively). They were also more likely to have higher grade and poorly differentiated breast tumors (*P* = 0.029 and 0.019, respectively). For the relationship with markers, compared to the low ADI group, high ADI group had higher median levels of allostatic load (*P* = 0.046) and lower median levels of global DNA methylation (*P* < 0.001). Compared to their counterparts, those from the high ADI group were 20% more likely to have increased allostatic load and 51% less likely to have increased levels of global DNA methylation. In summary, we observed that levels of allostatic load and global DNA methylation are influenced by neighborhood disadvantage among breast cancer patients.

## Introduction

Breast cancer is the most common malignancy among females and the leading cause of death worldwide^1^. Emerging evidence has shown that living in a disadvantaged neighborhood has been associated with a later stage of diagnosis, suboptimal treatment, and lower survival in women with invasive breast cancer^2–5^. However, how neighborhood disadvantage may biologically contribute to adverse breast cancer outcomes is still unclear. Emerging evidence has suggested that accelerated biological aging may serve as the critical player linking neighborhood disadvantage and aging-related chronic diseases, which may also contribute to poorer outcomes among breast cancer patients. Living in a disadvantageous neighborhood may lead to poor health behaviors^6,7^, increased toxicant exposures^8^, and lack of access to health services^9^. In addition, living in a deprived neighborhood can also lead to increased discrimination and segregation^10^. All of which above may result in increased chronic stress^11–14^, provoke biological weathering in endocrine and inflammatory systems, accelerate biological aging, and consequently result in an increased risk of aging-related diseases^15,16^. Among breast cancer patients these factors may translate into aggressive disease characteristics, poorer clinical outcomes, reduced quality of life, and shortened survival.

Though advanced age is one of the main risk factors for breast cancer, such a hypothesis has not been tested in breast cancer yet. It has been well-documented that poor healthy behaviors (e.g., obesity and being physically inactive) are associated with an increased risk of breast cancer and adverse clinical outcomes^17,18^. In addition, laboratory evidence has shown that exposure to environmental toxins, particularly endocrine-disrupting chemicals (e.g., bisphenol A (BPA) and dioxins), can promote the proliferation of breast cancer cells^19^. Our study found that increased allostatic load, a metabolic index related to chronic stress, is also associated with increased odds of having aggressive breast tumors among breast cancer patients^20^. Thus, it is very likely that biological aging may represent a meaningful biological link between the neighborhood characteristics and adverse breast cancer outcomes.

In this study, we evaluated the relationship between neighborhood disadvantage and markers of biological aging among breast cancer patients. The included markers were allostatic load, telomere length, and global DNA methylation in leukocytes, all of which are associated with breast cancer outcomes^20–22^. The study population included 906 women with newly diagnosed stage I to III breast cancer at M.D. Anderson from 2013 to 2018. Our hypothesis stated that living in the disadvantaged neighborhood (assessed using the Area Deprivation Index (ADI)) would be associated with accelerated indicators of biological aging among breast cancer survivors.

## Materials and methods

### Study population

The study population was derived from a breast cancer epidemiological study beginning in 2012^23^. Participants were patients at The University of Texas M. D. Anderson Cancer Center (Houston, TX) with newly diagnosed (defined by the presence of malignant breast epithelial cells) and histologically confirmed stage I to III (by microscopic analysis and molecular subtype) breast cancer. All patients were residents of the Greater Houston area. Blood samples were drawn before any cancer treatment. Written informed consent was obtained from each study participant. Self-reported ethnic background was used to define race and ethnicity. The Institutional Review Boards approved this study of M.D. Anderson Cancer Center and all study participants provided written informed consent before the baseline interview.

### Area deprivation index (ADI)

In this study, we used the area deprivation index (ADI) from the Neighborhood Atlas to assess the levels of neighborhood disadvantage^24^. The 2019 ADI (v3.1) was constructed using the 2015–2019 5-year estimates from the US Census American Community Survey. The description of Neighborhood Atlas and ADI can be found at https://www.neighborhoodatlas.medicine.wisc.edu/. A neighborhood is defined as a Census Block Group. A census block is the smallest geographic unit used by the United States Census Bureau to tabulate 100-percent data. A census block group comprises a set of blocks that average 100 population and the smallest unit with detailed demographic-economic characteristics. Thus, compared to the traditional neighborhood, the Census block group is more informative. The ADI provides the rankings of neighborhoods by a socioeconomic disadvantage in a region of interest. It includes factors for the theoretical domains of income, education, employment, and housing quality. This study used the national percentile rankings at the block group level from 1 to 100. The percentiles are constructed by ranking the ADI from low to high for the nation and grouping the block groups/neighborhoods into bins corresponding to each 1% range of the ADI. A block group with a ranking of 1 indicates the lowest level of "disadvantage" within the nation, and an ADI with a scale of 100 indicates the highest level of "disadvantage."

### Allostatic load

The construction of allostatic load was described extensively in our prior publication^20^. In brief, data were collected from interviews, medical records, and laboratory assays. We used a total of 17 factors to construct the allostatic load score, including systolic (SBP) and diastolic blood pressure (DBP), high and low-density lipoprotein cholesterol (HDL and LDL), total cholesterol, triglycerides, waist circumference, body mass index (BMI), glucose, hemoglobin A1C (HbA1C), albumin, estimated glomerular filtration rate (eGFR), creatinine, C-reactive protein (CRP), Interleukin-6 (IL-6), rest heart rate (RHR), and the history of taking medication to control metabolic diseases and hypertension.

### Telomere length and telomerase activity in leukocytes

The method for quantifying telomere length in leukocytes is described in our previous publication^25^. The real-time quantitative polymerase chain reaction (PCR) method was applied to measure leukocyte telomere length. Briefly, the ratio of the telomere repeat copy number (T) to the single gene (human globulin) copy number (S) was determined for each sample. The derived T/S ratio was proportional to the overall telomere length.

### Global DNA methylation in leukocytes

The method for quantifying global DNA methylation in leukocytes is described in our previous publication^26^. 5-Methylcytosine (5-mC) in blood leukocyte DNA was used as the marker of global DNA methylation. 5-mC was measured by the 5-mC DNA ELISA Kit (Zymo Research) according to the manufacturer’s instructions.

### Statistical analysis

A total of 961 breast cancer patients were eligible for the study. However, 55 were excluded due to missing data on critical epidemiological (demographics and healthy behaviors) and clinical variables (estrogen receptor (ER) status, tumor stage, and grade). So, the final study population included 906 breast cancer cases. ADI and biomarker data were available for all 906 patients. We used 50 percentiles of ADI at the national level as the cutoff to divide the study population into two groups: low ADI group (< 50%) and high ADI group (≥ 50%). First, we evaluated whether demographics (age, race, education, marital, and BMI), health behaviors (cigarette smoking, alcohol drinking, and physical activity), and tumor characteristics (ER status, tumor stage, and tumor grade) differed between low and high ADI groups. The adverse outcomes included ER-, stage III, and poorly differentiated tumor grade. The Student's test was used for continuous variables, and the Chi-Square test was used for categorical variables. Then, we assessed whether the levels among the markers of biological aging differed between low and high ADI groups using the Wilcoxon rank-sum test. We further categorized each maker of biological aging into a categorical variable (high vs. low) using median levels of each marker in the low ADI group as the cutoff point. To further clarify the association between the ADI group and markers of biological aging, we applied logistic regression analysis with the adjustment of demographic factors in model 1 and demographic and healthy lifestyle factors in model 2. Potential interactions between biological aging-related biomarkers with covariates were assessed. Odds ratios (ORs) and 95% confidence intervals (CI) were calculated in logistic regression analysis. In the exploratory analysis, we applied the Sobel test to assess whether and to what extent markers of biological aging mediated the association between ADI with tumor stage and grade. All reported P values were two-sided, and *P* < 0.05 was considered statistically significant. All analyses were performed using SAS v9.4 (SAS Institute).

### Ethical approval

All procedures performed in this study were approved by the Institutional Review Board at M D Anderson Cancer Center and in accordance with the ethical standards of 1964 Helsinki declaration and its later amendments or comparable ethical standards.

### Informed consent

Written informed consent was obtained from all participants.

## Results

This study included a total of 906 breast cancer cases. We did not observe any patients living in the most disadvantaged block groups from 81 to 100%. Five hundred forty-five were categorized into the low ADI group, and 361 were classified into the high ADI group. Table 1 illustrates the distributions of demographics, healthy behaviors, and tumor characteristics by ADI groups. Compared to those in the low ADI group, those in the high ADI group were younger (mean: 55.6 vs. 57.2 years old, *P* < 0.001), more likely to be non-Hispanic Black (25.21% vs. 12.48%, *P* < 0.001), and less likely to have a college degree (27.15% vs. 34.13%, *P* < 0.001). No significant difference was observed for marital status (*P* = 0.342). Regarding health lifestyle factors, those from the high ADI group were marginally more likely to have obesity than those from the low ADI group (34.07% vs. 28.25%, *P* = 0.080). However, physical activity, alcohol consumption, and tobacco use did not differ between high and low ADI groups. For tumor characteristics, there was a greater percentage of stage III diagnoses (38.23% vs. 31.19%, *P* = 0.029) and a greater percentage of poorly differentiated grade (55.12% vs 46.42%, *P* = 0.019) among those in the high ADI group relative to the low.

**Table 1:** Demographics, healthy behaviors, and tumor characteristics by ADI groups.

Baseline characteristics	Low ADI	High ADI	*P* value
N (%)	545 (60.15)	361 (39.85)	
ADI Range	≤ 50%	> 50%	
Age at diagnosis (years), mean (SD)	57.2 (1.7)	55.6 (1.9)	< 0.001
**Race**			
Non-Hispanic blacks	68 (12.48)	91 (25.21)	
Non-Hispanic whites	408 (74.86)	238 (65.93)	
Mexican Americans	69 (12.66)	32 (8.86)	< 0.001
**Education**			
Less than college	204 (37.43)	151 (41.83)	
Some college/associate degree	155 (28.44)	112 (31.02)	
College degree or above	186 (34.13)	98 (27.15)	0.085
**Marital status**			
Never married, separated, divorced, or widowed	265 (48.62)	188 (52.08)	
Married/Living with partner	280 (51.38)	173 (47.92)	0.342
**BMI**			
Normal weight	96 (17.61)	48 (13.29)	
Overweight	295 (54.13)	190 (52.63)	
Obese	154 (28.25)	123 (34.07)	0.080
**Physical Activity**			
High	114 (20.92)	84 (23.27)	
Medium	157 (28.81)	86 (23.82)	
Low	274 (50.28)	191 (52.91)	0.240
**Alcohol Consumption**			
Never drinker	145 (26.61)	90 (24.93)	
Former drinker	260 (47.71)	184 (50.97)	
Current drinker	140 (25.69)	87 (24.10)	0.630
**Tobacco Use**			
Never smoker	280 (51.38)	190 (52.63)	
Former smoker	212 (38.90)	135 (37.40)	
Current smoker	53 (9.72)	36 (9.97)	0.901
**ER status**			
ER +	391 (71.74)	245 (67.87)	
ER −	154 (28.26)	116 (32.13)	0.212
**Tumor stage**			
I/II	375 (68.71)	223 (61.77)	
III	170 (31.19)	138 (38.23)	0.029
**Tumor grade**			
Well differentiated	125 (22.94)	60 (16.62)	
Moderately differentiated	167 (30.64)	102 (28.25)	
Poorly differentiated	253 (46.42)	199 (55.12)	0.019

Next, we investigated the association between the ADI group and markers of biological aging. Compared to the low ADI group, median levels of allostatic load were statistically significantly higher in the high ADI group (*P* = 0.046) (Table 2). In addition, median global DNA methylation levels were statistically significantly lower in the high ADI group compared to the low ADI group (*P* = 0.035 and < 0.001, respectively). ADI was not associated with telomere length. We further categorized each marker into two categories (low and high) based on the median levels of each biomarker in the low ADI group. In the logistic regression analysis, we found that breast cancer patients from the high ADI group had 1.20-fold increased odds of having high allostatic load (OR = 1.35, 95%CI: 1.09, 1.74) and had 51% decreased odds of having high global DNA methylation (OR = 0.40, 95%CI: 0.27, 0.60) after the adjustment of demographic variables (e.g., age of diagnosis, race, education, and marital status) (Table 3). In further adjusting healthy behavioral factors (e.g., BMI, physical activity, alcohol drinking, and tobacco use), the significant associations remained (allostatic load: OR = 1.20, 95%CI: 1.02, 1.87; global DNA methylation: OR = 0.49, 95%CI: 0.33, 0.65). No significant association was observed between high telomere length and the ADI group.

**Table 2:** Comparison of biological aging related biomarkers by ADI groups.

Biomarkers, median	Low ADI (n = 545)	High ADI (n = 361)	*P* value
Allostatic load	7.21	7.53	0.046
Telomere length	1.12	1.08	0.085
Global DNA methylation	3.32	2.86	< 0.001

**Table 3:** Association between biological aging related biomarker category and ADI groups.

Biomarkers	Low ADI, N (%)	High ADI, N (%)	ORs* (95% CI)	ORs^#^ (95% CI)
**Allostatic load**				
Low	277 (50.83)	163 (45.15)		
High	268 (49.17)	198 (54.85)	1.35 (1.09, 1.74)	1.20 (1.02, 1.87)
**Telomere length**				
Low	275 (50.46)	191 (52.91)		
High	270 (49.54)	170 (47.09)	0.80 (0.61, 1.15)	0.84 (0.65, 1.19)
**Global DNA methylation**				
Low	268 (49.17)	245 (67.87)		
High	277 (50.83)	116 (32.13)	0.40 (0.27, 0.60)	0.49 (0.33, 0.65)

*. Adjusted by age at diagnosis, race, education, and marital status.

^#^. Adjusted by age at diagnosis, race, education, marital status, BMI, physical activity, alcohol drinking, and tobacco use.

Finally, using the mediation analysis, we examined the potential mediating role of higher allostatic load and global DNA methylation on the association between ADI group with tumor stage and grade (Table 4). Increased global DNA methylation was found to mediate 6.52 and 7.98% of the association between higher ADI group with grade III and poorly differentiated tumor grade (*P* = 0.037 and 0.023). No significant effect was observed for allostatic load.

**Table 4:** Mediating analysis to assess the role of biological aging markers mediating the association between ADI with tumor stage and grade.

Biomarkers	Tumor stage	*P* value	Tumor grade	*P* value
Allostatic load	4.47%	0.162	5.82%	0.149
Global DNA methylation	6.52%	0.037	7.98%	0.023

## Discussion

To our best knowledge, this is the first study to assess the relationship between neighborhood disadvantage and markers of biological aging among breast cancer patients. In the study, we reported that breast cancer patients who lived in the area with high ADI had higher median allostatic load levels and lower median global DNA methylation levels. In addition, global DNA methylation was found to mediate 6.52 and 7.98% of the association between ADI with stage III and poorly differentiated tumor grade (*P* = 0.037 and 0.023). The results from this study shed light on the importance of biological aging in linking neighborhood disadvantage with aggressive breast tumor characteristics and other adverse clinical outcomes.

During the aging process, epigenetic changes, including global DNA hypomethylation and hypermethylation at regions of CpG island, are commonly observed^27^. Such changes in the epigenetic landscape may contribute to aging by adversely affecting genomic stability and gene regulation. In this study, we found a statistically significant association between high ADI and decreased levels of global DNA methylation. The possible pathways underlying the are likely to be multifactorial. For example, living in a disadvantaged neighborhood is known to have lower levels of physical activity and a higher likelihood of being overweight or obese. In a meta-analysis of 24 studies, a trend between higher levels of physical activity and higher levels of global DNA methylation was observed^22^ and subsequently led to a suggestive association with a reduced breast cancer risk. Furthermore, living in a disadvantaged neighborhood may have increased exposure to certain endocrine disruptors (e.g., BPA and dioxins)^19^. It has been known that exposure to endocrine disruptors influences DNA methylation globally and CpG site specifically^28^. Interestingly, breast tissue is particularly susceptible to the effects of endocrine disruptors during the development period as well as adulthood^29^. In addition, chronic stress is known to induce persistent changes in global DNA methylation and gene expression^30^.

The relationship between neighborhood disadvantage and global DNA methylation has rarely been studied. Only one study has been found in the literature. Coker et al. found that pregnant women living in neighborhoods with the highest poverty levels gave birth to infants with higher cord blood LINE-1 methylation compared with pregnant women living in the lowest level of poverty^31^. On the contrary, several studies have investigated the gene or CpG site-specific methylation and its relationship with neighborhood disadvantage. For example, in the Multi-Ethnic Study of Atherosclerosis (MESA) study, neighborhood characteristics have been found to influence DNA methylation of genes involved in stress response and inflammation^32^. More interestingly, using newly developed epigenetic clocks based on DNA methylation, Lawrence et al. recently reported that residing in a neighborhood with a higher deprivation index had higher epigenetic age acceleration estimated by Hannum, PhenoAge, and GrimAge clocks^33^.

The positive association between high ADI and increased allostatic load is consistent with previous reports^34^. In a scoping review of 14 studies, most studies (*n* = 12) reported a significant association between neighborhood deprivation and allostatic load, even after adjustment for confounding variables such as individual socioeconomic status, gender, and age^34^. In another study using data from three cohort studies in European countries, a statically significant trend of increasing allostatic load with the increase of area deprivation (*P* < 0.001) was reported^35^. To dissect the potential pathways that might help explain the relationships between ADI with global DNA methylation and allostatic load, we also assessed adjustment for healthy behaviors (e.g., tobacco use, alcohol drinking, obesity, and physical inactivity) influenced the association. Though the adjusted associations attenuated slightly, the significant association has remained. Clearly, those healthy behaviors didn’t fully account for the significant association. It is very likely that other pathways in addition to health-related behaviors contribute to the observed associations.

Unlike previous studies, we didn’t observe the significant association between telomere length with neighborhood disadvantage^36^ and several neighborhood characteristics measured subjectively and objectively^37–40^. In a previous study using the data from the 1999–2002 National Health and Nutrition Examination Survey, neighborhood deprivation was inversely associated with telomere length among individuals living in neighborhoods with medium neighborhood deprivation index (NDI) (*P* = 0.0005) and high NDI (*P* = 0.003)^36^. However, only medium NDI (*P* = 0.009) was associated with shorter telomere length among women. One significant discrepancy between our study and those studies is the study population. Unlike those studies, our study population is consistent with women diagnosed with breast cancer. Telomere length has dual role in cancer development. Though critically short telomeres may lead to chromosomal degradation, end-to-end fusion, and abnormal recombination, processes involved in cancer development, cells with longer telomeres may favor delayed cell senescence, thus increasing the chance of developing chromosomal instability and genetic aberrations, eventually leading to carcinogenic transformation^41^. In cancer epidemiological studies, both longer or shorter telomere length has been associated with an increased risk of breast cancer^42^.

A few studies have examined the relationship between neighborhood disadvantage and breast tumor characteristics. In three Appalachian states, area deprivation is strongly associated with the disparity in the later stage of breast cancer. The most deprived counties had a 3.31 times greater rate of LSBC compared to the least deprived^43^. In a recent publication by Babatunde et al., a higher social deprivation index was associated with a distant stage at diagnosis (OR = 1.06, 95% CI: 1.02–1.10) among breast cancer patients^44^. We have similar findings in our study. Furthermore, we found that the mediation effect of global DNA methylation between higher ADI group with grade III and poorly differentiated tumor grade. Our results highlight the role of global DNA methylation in linking neighborhood disadvantage and adverse tumor characteristics among breast cancer patients.

There are several limitations to this study. The study design is cross-sectional, and none of the breast cancer patients included in this study live in the area with an ADI of over 80%. This may limit our statistical power to detect the significant association. It may also reduce our ability to interpret and generalize the findings. There are many different area-based deprivation measures developed and available to public health scientists. Other measurements may produce different results. However, nearly all of them have included the same or similar factors in income, education, employment, and housing quality extracted from the Census data. Thus, we believe the overall results from those measures will be similar to the levels of neighborhood disadvantage. The selection of ADI over other measures is mainly because this ADI measure has been frequently used in the scientific community, which demonstrates its high validity. Though the study has included non-Hispanic Black and Mexican American breast cancer patients, the number is modest. Thus, we may not have adequate power to assess whether the association differs by race. Additionally, markers of biological aging were measured only once. A single measurement may not reflect the dynamic nature of the relationship between neighborhood disadvantage and biological aging. Finally, we don’t have a validation population included in this study. Nevertheless, the considerable strengths of our study outweigh the limitations. Findings from this study contribute important knowledge on how neighborhood disadvantage may affect breast cancer outcomes through accelerated biological aging. Additional research, particularly addressing the limitations mentioned above, is needed to validate our findings, and further clarify the association.

## Data Availability

The datasets used and/or analyzed during the current study are available from the corresponding author on reasonable request.
